# Expanding voter registration to clinical settings to improve health equity

**DOI:** 10.1111/1475-6773.14218

**Published:** 2023-08-14

**Authors:** Brooke Stanicki, Madeline Grade, Julianna Pacheco, Laura Dugan, Alexandra Salerno, Alister Martin

**Affiliations:** ^1^ Temple Lewis Katz School of Medicine Temple University Philadelphia Pennsylvania USA; ^2^ Department of Emergency Medicine University of California San Francisco San Francisco California USA; ^3^ Department of Political Science University of Iowa Iowa City Iowa USA; ^4^ Civitech Austin Texas USA; ^5^ Harvard Medical School Mass General Emergency Medicine Boston Massachusetts USA

*Voting* plays a critical role in the allocation of social and structural resources that are associated with population health and health equity. Even the medical community—which has long focused on the biological determinants of health—has recognized the fundamental role of social–political factors, such as voting. For instance, on June 14, 2022, the American Medical Association passed Resolution 422, acknowledging that “voting is a social determinant of health and significantly contributes to the analyses of other social determinants of health as a key metric”.^1^ The County Health Rankings^2^ has followed suit finding that civic participation is a key component of building power to improve health (see their 2023 National Report Findings).

Early evidence suggests that there is indeed a correlation between engagement in the democratic process and improved health outcomes, partially due to the downstream effects of electing representatives that vote for community interests. The health of a community has multi‐faceted influences, but is particularly sensitive to policies regarding the allocation of social assistance, education, child development, housing, and social services. Ultimately, if a community lacks a political voice, they lose influence over the elected officials who vote for these policies, exacerbating subsequent inequities.

As one example, counties with restrictive voting laws—such as voter identification laws and limited polling hours, had higher COVID‐19 case and mortality rates than counties with expansive voting laws.^3^ Restrictive electoral laws have historically disenfranchised racial/ethnic minorities,^4^ suggesting that these laws are a byproduct of systematic racism with the potential to influence health disparities across race and ethnicity. A study by Cottrell et al.^5^ finds that incarceration—which disproportionately affects African Americans—contributes to premature death, which in conjunction with felon disenfranchisement laws results in some legislative districts having African American disenfranchisement rates between 20% and 40%. This results in millions of African Americans literally “missing” from their communities. Rodriguez et al.^6^ estimate that excess mortality reduced the voting age population among African Americans voting in the 2004 presidential election by 1.7 million people. The lost votes affected the presidential outcome and—if included—likely would have reversed several senate and gubernatorial elections.^6^ This research, even in its infancy, suggests that disenfranchisement and inaccessibility in American democratic processes not only uphold political inequities but also contribute to worsening health disparities, particularly across race/ethnicity, which then further dampens political voice among these groups.

## 1 VOTER REGISTRATION AS A BARRIER TO HEALTH EQUITY

Voter registration is a particularly important barrier to achieving political equity, and by extension, health equity. The complexities ingrained into the American voter registration system have persisted as a major reason why turnout in the United States trails other democracies.^7^ Registration in the United States is not automatic; citizens must actively register to vote. State‐to‐state and even county‐to‐county differences in same‐day voter registration, open polling hours, absentee voting procedures, and the opportunity for online voter registration among other policies results in a patchwork of registration laws.^8^ The inconsistencies in this system mean that in some states, registering to vote is easily accessible, while in others, the registration process places a substantial burden on citizens to understand when, how, and where to register to vote.^9^

Previous work has found that the costs associated with registration fall disproportionately on historically disenfranchised populations, including voters from low‐income communities and racial and ethnic minorities who are more likely to work multiple jobs, work during polling place hours, lack access to childcare and transportation, and have higher rates of illness and disability.^10^, ^11^ These factors and others contribute to the consistently lower voter registration rates for Black (69%), Asian (63.8%), and Hispanic (61.1%) voters compared to Non‐Hispanic White (75%) voters.^12^ Similarly, persons belonging to households earning under $30,000 have significantly lower rates of voter registration (under 66%) compared to those in households earning over $75,000 (over 85%).^12^ For policymakers interested in expanding the electorate and reducing health inequalities, making registration more accessible is imperative.

## 2 THE DEPARTMENT OF MOTOR VEHICLES AS REGISTRATION SITES PERPETUATES INEQUALITIES

A main goal of the National Voter Registration Act (NVRA) of 1993, colloquially referred to as the “Motor Voter Law”,^13^ is to increase registration and turnout by reducing the costs associated with voter registration and to make the registration process more uniform across jurisdictions. Section 5 of the NVRA requires voter registration at all state Department of Motor Vehicles (DMV) locations, and since its passing, DMV voter registration has become central to registration nationally. Other sections of the law are currently not utilized on a wide scale, but show promise. Section 7 of the NVRA, for instance, reads, “each State shall designate as voter registration agencies…all offices in the State that provide public assistance; and all offices in the State that provide State‐funded programs primarily engaged in providing services to persons with disabilities”.^14^ The law also cites public libraries, public schools, state colleges, universities, and unemployment compensation offices as other agencies that can be designated as registration sites under the discretion of the states. Statewide initiatives with similar goals of expanding the electorate also exist, including laws in 23 states for automatic voter registration (AVR) for residents who interact with the DMV.^15^

These efforts are a step in the right direction, yet research consistently shows the NVRA did not dramatically expand or diversify the electorate^16^ and may have even exacerbated inequalities.^17^ One reason for the relative failure of the NVRA is the lack of compliance of Section 7, which as we noted above, requires public assistance agencies to provide voter registration services to every person applying for or renewing government benefits. In addition, Michener^18^ finds that *race* is a pivotal factor of when states incorporate low‐income policy beneficiaries via Section 7. State compliance is lower in states where non‐whites are less active in electoral politics as well as where African‐Americans comprise a greater share of the state population.^18^

The NVRA's minimal effects are also likely because of the drawbacks of using DMVs as primary registration sites to incorporate marginalized populations. DMV locations can be limited in number and unevenly dispersed, particularly as population density changes over time in a given municipality. For example, in Pennsylvania alone, there are 9,300 polling places in the state compared to the 71 DMV offices, with only four offices serving Philadelphia, the 5th largest US city by population.^19^ About 14% of US citizens do not drive or possess a driver's license and therefore do not interact with the DMV (Highway Statistics Series^20^). These individuals are more likely to be people of color and low income.^21^ People of color are also more likely to have their licenses suspended due to unpaid traffic tickets, with Black males comprising the majority of all suspended licenses.^22^ Finally, DMVs are at the whim of state budgetary decisions. As one example, in 2015, Alabama closed 31 DMV offices—located in disproportionately poor, rural, African American communities—due to budget constraints.^23^ Given that these are the same groups that have disproportionately low voter registration rates, there is substantial evidence to the claim that unequal access to the DMV offices contributes to the inability of DMV‐based voting reforms to sufficiently impact marginalized groups.^24^

## 3 THE CASE FOR EXPANDING VOTER REGISTRATION TO HEALTHCARE SITES

In looking for additional ways to improve political equity, President Biden signed Executive Order 14019 “Promoting Access to Voting” on March 7, 2021. EO14019 requires federal agencies to evaluate their voting efforts, submit a strategic plan outlining how they can better promote political engagement, and coordinate actions across levels of government (Exec Order No. 14019, 2021). Given that hospitals provide public assistance and disability services, these locations are appropriate for voter registration sites under the provisions of the NVRA and EO14019. These settings also have the potential to provide more equitable and convenient access to groups with low rates of voter registration compared to DMVs. Hospital emergency departments treat disproportionately high rates of non‐Hispanic Black patients and patients who either do not have insurance or are on Medicare, likely in part because these groups are more likely to report utilizing acute care facilities for routine care.^25^ Acute care settings are also more likely to see people from low‐income households, as those making under $35,000 per year are over six times as likely to visit an emergency department for non‐emergencies than those making over $100,000.^26^ This points to structural racism and insurance barriers in healthcare that are currently being stop‐gapped by hospital emergency departments. Although there is still significant work to be done in addressing these issues, their role in serving underserved populations makes them a potential opportunity location for helping these same patients gain political power.

Community healthcare centers (CHCs), including organizations designated as Federally Qualified Health Centers (FQHCs) and Health Center Program look‐alikes (LALs), are also included under EO 14019. Serving more than 28 million patients, CHCs are especially equipped to empower politically disadvantaged individuals. Roughly 62% of patients are members of racial and ethnic minority groups, 90% are at or below the 200% federal poverty level, and 24% are best served in a language other than English.^27^ CHCs are also uniquely situated to promote community empowerment. They are located in medically underserved areas; they offer comprehensive healthcare services; they are open to anyone regardless of insurance status or ability to pay; and they have patient‐majority governing boards.^27^

Healthcare sites, whether emergency departments or CHCs, often create the settings necessary for highly successful mobilization efforts due to what political scientists refer to as relational conditions. Healthcare sites are central parts of communities, sharing a past and being invested in the implied future of the areas they serve. Community care is often responsive to constituent concerns and delivered in a manner that meets the patient's biological, psychological, social, and emotional needs. There is also a long tradition of the democratization of health decision‐making dating back to the 1960s and 1970s whereby health professionals cooperate and negotiate with constituents to better serve the community.^28^ By sharing a past and implied future and by being open and responsive to constituents, healthcare organizations are likely to have better success at promoting civic involvement compared to DMVs, which tend to lack these relational conditions.

Individuals also tend to view doctors more positively compared to other professions. In a study by Patashnik et al.,^29^ when asked about what drives motivations of doctors, lawyers, grade school teachers, and members of Congress, respondents viewed doctors as more interested in helping people, more trustworthy, and as caring more (“about people like me”) compared to other groups. People also have more favorable opinions about medical associations compared to unions, business organizations, or health insurance organizations.

For these reasons, physicians and medical associations have a unique and powerful opportunity to influence voter registration and therefore political equity. Some healthcare‐based voter registration organizations have already mobilized, empowered by commitment and support from their communities, to start voter registration efforts in clinical settings.^30^ Non‐partisan organizations such as Vot‐ER and hospital‐specific drives like Massachusetts General's “MGH Votes” campaign have already started offering voting registration in clinical settings with promising early results.^30^ One clinician‐led, nonpartisan voter registration drive conducted within two FQHCs in the Bronx, New York successfully registered 89% of eligible voters in the 2012 election.^31^ Another nonpartisan voter registration effort targeted toward patients aged 18–22 found that 44% of participants who registered to vote in the primary care clinic voted in the 2018 midterm elections.^32^ The need for alternative voter registration sites was made particularly clear in 2020, when pandemic restrictions caused DMVs to close or become backlogged and created other significant barriers to in‐person registration methods.^33^ When asked, a majority of patients were receptive to the idea of registering to vote in a clinical setting.^30^

Our own analyses (details in the Appendix) support the notion that adding registration sites in healthcare settings can significantly reduce the costs associated with voter registration, particularly for marginalized populations. The addition of hospitals as voter registration sites (1) decreases the average distance traveled for residents in a diverse metropolitan area and (2) reduces the necessary service area for registration sites. In Philadelphia, residents in the far northern part of the county would have up to 6.5 m less to commute to register to vote when hospitals are included as potential registration locations; however, population density here is relatively low. Residents in the more densely populated areas would see smaller distance gains to potential registration locations, but these gains could have an outsized impact due to the challenges of travel through dense urban areas. Given that non‐White residents tend to reside in more densely populated areas, the gain in distance along with fewer public transportation transfers may confer even greater benefit to these groups. The reduction in service area size across the city could allow for less congestion at each voter registration site, further improving access. This is a key intervention in turning a healthy quality issue into a healthy equity issue.

## 4 A CULTURE OF HEALTH RESTS ON POLITICAL EQUITY: A CALL TO ACTION

A culture of health rests on political equity. One way to allow for disenfranchised populations and communities of color to be able to influence the public policies that directly impact them is to expand access to voter registration process. When it was passed, NVRA sought to address inequities and standardize national voter registration, but it has instead siloed voter registration into DMV locations. This practice fails to provide registration to those for whom the DMV is not accessible, which is predominantly people of color and those of low socioeconomic status. Healthcare centers, including community health centers and those that provide emergency department care, already offer public assistance programs, making them approved voter registration locations under the NVRA and EO14019. The American healthcare system is imperfect, as it is still grappling with its history of discriminatory practices and structural racism. However, many healthcare centers are not only often utilized by people of color, but also serve as community hubs, and have historically hosted successful mobilization efforts. Additionally, the physician role in society has cultivated a significant amount of trust, giving providers a unique and powerful position to encourage civic engagement. Ultimately, healthcare providers have an incredible opportunity to shape the communities where they practice and improve health outcomes for generations, without having to write a prescription or pick up a scalpel. To expand voter registration into healthcare settings is an investment in improving the political power of underrepresented populations, and therefore, is an investment in the health and well‐being of our communities.

## Figures and Tables

**FIGURE A1:**
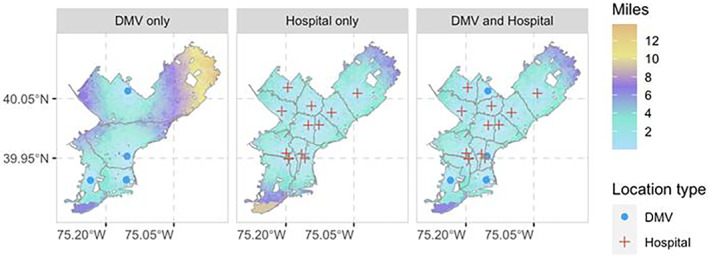
Drive distance mapped using 2020 census blocks in Philadelphia County, PA. Comparison of the drive distance if only DMVs were utilized for voter registration (left) compared to the service area size if only hospitals were utilized (middle) and if both hospitals and DMVs are utilized (right). If both DMVs and hospitals are utilized, all census blocks have a shorter drive distance to a voter registration site. [Color figure can be viewed at wileyonlinelibrary.com]

**FIGURE A2:**
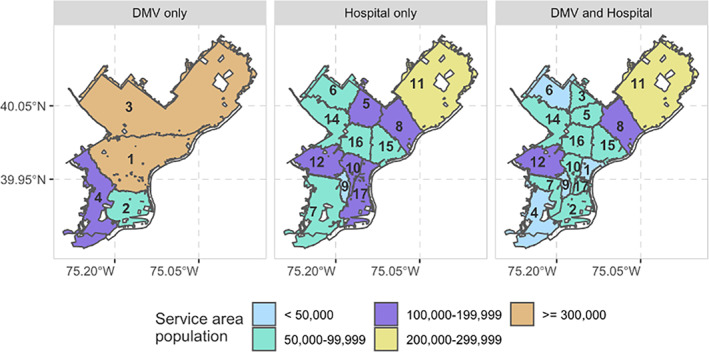
Potential voter service area populations mapped for DMV‐only registration, hospital‐only registration, and DMV and hospital registration. Locations 1–4 are the DMV locations and locations 5–17 are the hospitals. Each color represents the population size of the potential service area. With the inclusion of both DMVs and hospitals, service area population decreases for all service areas. [Color figure can be viewed at wileyonlinelibrary.com]

## APPENDIX A EXPANDING VOTER REGISTRATION TO CLINICAL SETTINGS TO IMPROVE HEALTH EQUITY

### A.1

Philadelphia is a diverse and heavily populated urban center with notable segregation in its population and limited number of DMVs.^12^ Philadelphia is a national healthcare leader, boasting locations of 36 community health and hospital systems including the University of Pennsylvania Health System, the Temple University Health System, and the Jefferson University Health System. There is also an opportunity to expand access to voter registration to disenfranchised groups. One in 10 adults—the majority who are members of minority groups, have low income, and are without health insurance—report using emergency departments for primary care in Philadelphia (City of Philadelphia, 2018). Does adding healthcare sites as voter registration locations affect travel distance for different subgroups of Philadelphia residents?

#### A.1. Data acquisition and preparation

There were four DMVs and 12 hospitals in the study area. Their addresses were first gathered using the Philadelphia Department of Transportation (PennDOT) website, * The Commonwealth of Pennsylvania. “Open Locations.” PennDOT Driver & Vehicle Services. Accessed October 2, 2022. https://www.dmv.pa.gov/Driver-Services/Pages/Open-Locations.aspx. the City of Philadelphia website (phila.gov), † Department of Public Health. “Covid‐19 Urgent Care Centers: Programs and Initiatives.” City of Philadelphia, November 18, 2020. https://www.phila.gov/programs/coronavirus-disease-2019-covid-19/covid-19-urgent-care-centers/. and the American Hospital Directory (AHD)^23^. 2020 Census blocks were downloaded from the Census Bureau FTP site for Philadelphia County. ‡ US Census Bureau. “American Community Survey Data FTP.” Census.gov, September 7, 2022. https://www.census.gov/programs-surveys/acs/data/data-via-ftp.html. A road network was obtained using the Humanitarian OpenStreetMap Team (HOT) Export Tool for Philadelphia County and the surrounding area. § OpenStreetMap Contributors. Hot export tool. OpenStreetMap Contributors. Accessed October 2, 2022. https://export.hotosm.org/en/v3/. Roads were converted into osm format and then added to a PostgreSQL database as a topological network. Registration locations were geocoded using Google's rooftop precision API and the ggmap R package. Census block center points were created and coded into the database using the centroid when it fell within a block. When it did not, they were placed as a point on the surface. In order to calculate distances, the shortest route along the road network from every Census block center point to the nearest potential registration location was determined using the withpointscost() function in the PostgreSQL pgrouting extension. This function is based on the Djikstra shortest path algorithm that uses edge weights (i.e., road segment distances) to find the shortest path between two points.

#### A.2. Travel distance

Travel distance data were compared across three groups: DMV only, hospital only, and both DMV and hospital. Mean distances were calculated as the mean distance from each Census block center point to the nearest registration location, whereas weighted mean distances were weighted by the total population in each block. Additionally, one‐way ANOVAs were performed to evaluate if the mean/weighted mean distances traveled from Census block center points to the nearest registration locations were significantly different between at least two analysis groups, and Tukey's HSD was applied to determine which specific groups significantly differed from each other.

#### A.3. Service area and race sub‐analysis

Service areas were defined as the geographic area serviced by a given potential registration location and were determined by grouping together Census blocks with the same closest registration location. For example, all Census blocks that had DMV 1 as the closest registration location in either the DMV only or DMV and hospital analyses were grouped into the same respective service area. The mean population, size, distance and weighted distance, and demographic breakdown were calculated for each service area for each analysis group and service area. The mean distance from Census block center point to the closest registration location, weighted by the population of each demographic in the given block, was also assessed by race and analysis group, and a two‐way ANOVA was performed to assess if race and/or analysis group affected this distance.

#### A.4. Results

Using hospitals alone or including hospitals with DMVs as potential registration locations decreased the distance to the nearest registration location, the total area that an individual registration location services, and the number of individuals that reside closest to that registration location compared to the current scenario of using DMVs alone. Under the current scenario (DMV only), residents primarily in the northern part of the county must travel long distances to reach a registration location. Switching to hospitals only would decrease this; although, it would concomitantly increase the distance to registration location for residents in the southern part of the county due to the lack of a hospital in the far south. Figure A2 shows that using both DMVs and hospitals results in reduced travel distances for residents in all parts of the county.

Regarding the change in distance traveled between analysis groups, there was a nearly 50% decrease on average when comparing DMVs only to hospitals only, with an additional 5% decrease in distance to closest registration location when both DMVs and hospitals were considered. Service area size and population decreased by 66% and 75%, respectively, when comparing DMVs only to hospitals only and DMVs only to DMVs and hospitals. Figure A2 shows the far north of the county saw a more than 6.5 mi absolute decrease in distance required to travel to a registration location when comparing the DMV only and DMV and hospital scenarios.

#### A.5. Service area

Service areas were smaller and serviced fewer people in the hospital only and DMV and hospital analysis groups relative to the DMV only group (Figure A1). When only DMVs were offered as registration locations, the number of people serviced by each site was high, particularly in the central and northern parts of the county where there were no DMVs, and the weighted mean distance from Census blocks within the service area to the registration location was much greater.

#### A.6. Differences by race

Non‐White residents were distributed throughout Philadelphia county with large concentrations in the central north and southwest, whereas the White population was concentrated in the northwest and northeast as well as on the southern border. White residents had to travel, on average, roughly 4.4, 2.0, and 1.9 miles to the closest registration location, respectively, for the DMV only, hospital only, and DMV and hospital analysis groups, whereas non‐White residents had to travel, 3.6, 1.8, and 1.6 miles, respectively. All demographics benefited from including hospitals along with DMVs as potential registration locations (i.e., 0.50–0.65 mi closer on average across all groups) with White and non‐White populations experiencing a similar improvement in distance from voter registration site (0.57 and 0.57 mi closer, respectively).
